# Mr. Churchill's Manuals

**Published:** 1846-04

**Authors:** 


### Art. XIV.

### MR. CHURCHILL'S MANUALS.

1. *A Manual of Physiology, including Physiological Anatomy; for the use of the Medical Student.* By William B. Carpenter, M.D., F.R.S., &c.
2. *A System of Practical Surgery.* By William Fergusson, Esq., F.R.S.E., Professor of Surgery in King's College, London, &c. Second Edition.—*London,* 1846. pp. 668.
3. *A Manual of Medical Jurisprudence.* By Alfred S. Taylor, F.R.S. Second Edition.—*London,* 1846. 8vo, pp. 704.

It has been our misfortune to have to turn the light of our critical countenance from so many books bearing the well-known sign and superscription of John Churchill, that we can afford to praise our worthy publisher, when he deserves it, without any danger of being accused of partiality. And, assuredly, when we look at the three splendid volumes now before us, and the others previously published on precisely the same plan,* which, under the humble name of Manual, comprise the most elaborate and complete treatises on their respective subjects—when we inspect their beautiful typography and incomparable woodcuts—and compare their extremely moderate price with what we know must have been the very large outlay requisite to bring them to such a state of perfection,—we must honestly admit that the obligations are mutual between the bibliopolist of Princes street and the members of the medical profession. If by their patronage he has waxed rich and portly, they, in their turn, are benefited by, and thus become sharers in, his prosperity. Nothing but a large capital could bring into the market works of this very expensive sort, and so rapidly one after another; and nothing but a most extensive sale could make the outlay of that capital remunerative, under the low price at which the volumes are sold. No doubt our good friend knows what he is about. We do not accuse him of any superhuman regard for the poverty of the medical exchequer. If he is liberal to his writers and liberal to his customers, it is a very possible case that he has found out that such liberality has no deteriorating effect on the treasury of Princes street. But be that as it may, we have so much reason to be satisfied with the result, as far as it concerns us, that we here give him public thanks for

* A Manual of Chemistry, by George Fownes, F.R.S.; The Anatomist's Vade Mecum, by Erasmus Wilson, F.R.S.; Elements of Natural Philosophy, by Golding Bird, M.D., F.R.S.

the positive benefit conferred on the medical profession by the series of beautiful and cheap Manuals which bear his imprint, and of the last published of which we now proceed to give an account.

### I. Dr. Carpenter's Physiology.

The multiplication of general treatises on physiology, especially of those intended for the medical student, is a cheering indication of the increased attention which is being paid to this department of medical science—a department whose importance is now coming to be acknowledged as universally as it was formerly overlooked. This little volume, we are informed by the author, owes its origin to a desire on the part of Mr. Churchill to add an elementary treatise on physiology to the series of student's manuals which he had previously issued; and the success of Dr. Carpenter's larger works naturally led him to press the execution of the task upon that gentleman, who would otherwise, he assures us in his preface, have been better pleased to devote his leisure time to the prosecution of original researches in his favorite department of study.

We are led by the preface to expect some degree of novelty in the plan of his treatise, that should render it something else than a mere abridgment of the author's larger works, which it must have otherwise almost necessarily become; and a glance over its pages will show in what this novelty consists. Ever anxious to impress his readers with the *principles* of the science he is expounding, and desirous to elucidate these by all the light to be derived from recent discoveries, Dr. Carpenter has devoted a large proportion of the early part of the volume to an account of the elementary parts of the human body—in other words, the primary tissues—including their structure, chemical composition, mode of growth and development, and their actions in the general economy. The following extract will show the conformity of his plan to that which would be adopted in any parallel case, and will also indicate that he has prosecuted it in accordance with the most advanced views of the present remarkable era in physiology—that in which the independent life of cells and their functional instrumentality have come to be recognised as the great fundamental facts in the vital actions of animals, as they have long been known to be in that of plants.

"In the investigation of the operations of a complex piece of mechanism, and in the study of the forces which combine to produce the general result, experience shows the advantage of first examining the component parts of the machine—its springs, wheels, levers, cords, pulleys, &c.—determining the properties of their materials, and ascertaining their individual actions. When these have been completely mastered, then attention may be directed to their combined actions; and the bearing of these combinations upon each other, so as to produce the general result, would be the last object of study. This seems to be the plan which the student of physiology may most advantageously pursue in the difficult task of making himself acquainted with the operations of the living fabric, and with the mode in which they concur in the maintenance of life. We should first examine the properties of the component materials of the structure in their simplest form; these he will find in its nutrient fluids. He may next proceed to the simplest forms of organized tissue, which result from the mere solidification of these materials, and whose properties are chiefly of a mechanical nature. From these he will pass to the consideration of the structure and vital actions of those tissues that consist chiefly of cells, and will investigate the share they take in the various operations of the economy. Next his attention will be engaged by the tissues produced by the transformation of cells; of which some are destined chiefly for affording mechanical support to the fabric, and others for peculiar vital operations. And he will then be prepared to understand the part which these elementary tissues severally perform in the more complex organs. A due knowledge of these elementary parts, and of their physical, chemical, and vital operations, is essential to every one who aims at a *scientific* knowledge of physiology. True it is that we may study the *results* of their operations without acquaintance with them; but we should know nothing more of the *working* of the machine than we should know of a cotton mill, into which we saw cotton wool entering, and from which we saw woven fabrics issuing forth; or of a paper-making machine, which we saw fed at one end with rags, and discharging hot-pressed paper, cut into sheets, at the other. The study of these results affords, of course, a very important part of the knowledge we have to acquire respecting the operations of the machine; but we could learn from them very little of the nature of the separate processes effected by it; still less should be prepared, by any disorder or irregularity in the general results, to seek for and rectify the cause of the disturbance in the working of the machine, by which the abnormal result was occasioned." (p. 94.)

It is a great advantage of the general view of the elementary operations of the animal economy, which is thus presented, that the author is thereby enabled to carry forward his description of the several functions (which occupies the second part of the book) in what we cannot help regarding as their natural order; namely, commencing with the ingestion of food, tracing its gradual conversion into the fabric of the body, and its final disintegration; then proceeding with the reproductive operations; and closing with the functions of animal life, the maintenance of which is so completely the final cause or purpose of the preceding. The objection felt by the author to the adoption of this course in his human physiology—namely, the intervention of the nervous system in almost every one of the organic functions—is obviated by the previous explanation of the general operations of the nervous apparatus, which is contained in the first part.

Among the novelties in this volume, which indicate the progress of the author's general views, we may advert to his description of the ultimate composition of the muscular fibrilla, in which he seems to us to have advanced considerably both upon Mr. Bowman and upon Mr. Erasmus Wilson. He describes and figures the fibrilla as composed of a series of short cylindrical cells, laid end to end, the cavities of which, being dark, (probably in consequence of containing some highly-refractive material,) give rise to the series of equidistant dark spots in the fibril; whilst the intervening light spaces are made up by the contiguous walls of each pair of cells, which are separated by a distinct transverse line, equidistant between the dark spots. The white portion of the fibril is stated by Dr. Carpenter to present itself, not merely *between*, but also *around* the dark spots; which seems almost conclusive as to its being the cell wall. The figure given by Dr. Carpenter closely resembles one given by Dr. Goodfellow in the short-lived 'Physiological Journal;' but his explanation is altogether different, and to our minds much simpler. According to Dr. Goodfellow, the dark points are the "sarcous elements" of Mr. Bowman, imbedded in a tube of sarcolemma, which possesses transverse septa at intervals; whilst by Mr. Erasmus Wilson, in his 'Vade Mecum of Anatomy,' the strange mistake (for such we must regard it) is made of describing the dark spots and the intervening light spaces as *each* consisting of cells, of which he is obliged, therefore, to suppose two distinct kinds to exist—a pair of light cells (separated by the transverse line above mentioned) being interposed between each pair of dark ones. To those who are acquainted with vegetable structures it may be sufficient to state that the fibril, in Dr. Carpenter's eyes, has precisely the characters of a minute conferva; and that the phenomenon of contraction consists in just such a change of form of the individual cells as that which occurs in the contraction of the irritable tissues of plants. He thus extends his previous generalization. Having shown that all the essential parts of the organic nutritive functions of animals are carried on, like the corresponding actions of plants, by the agency of cells, and having pointed out the strong reason there is for the belief that, wherever changes in the nervous system originate, their immediate instruments are of this very same character, he is enabled to show that *all* the active vital changes in the animal body may be reduced to this one great principle of *cell-life.*

We observe that in treating of the process of sanguification, the spleen, the supra-renal capsules, and the thymus and thyroid glands are included with the glandulæ of the absorbent system in the general assimilating apparatus; the cells of their parenchyma being supposed to withdraw certain materials from the blood, and then to restore them, after having exercised upon them an elaborating action. This view, for the grounds of which we must refer our readers to the book itself, is confirmed in a very striking manner by the interesting embryological researches of Mr. J. Goodsir, very recently communicated to the Royal Society; it having been shown by that gentleman that the three last of the above-named organs *are persistent portions of the original germinal membrane or blastoderma;* which is the sole organ by which the assimilation of the materials supplied by the yolk is effected during the earlier periods of development, and which thus leaves behind it (so to speak) some fragmentary portions to aid in carrying on the process during the earlier period of extra-oval life.

Various practical hints are interspersed through the pages of this beautiful volume, which may help, we trust, towards the erection of that new temple of Therapeia in which we call upon all our readers, young and old, to "bear a hand;" and we may point to the 'General Summary of the Excreting Processes,' (pp. 437-40,) as of peculiar value in this respect—not from any novelty in the ideas put forward, but as being invested with a definite and tangible shape, very different from those of the old humoral pathology, and much more likely to exert an advantageous influence on the treatment of disease.

The author has shown singular skill in preserving so marked a line of distinction between the present Manual and the 'Principles of Physiology' previously published by him. They are both on precisely the same subject; but the one is neither a copy, nor an abstract, nor an abridgment of the other. In one thing, however, they are exactly alike—in their general excellence, and in their perfect adaptation to their respective purposes. The reputation of Dr. Carpenter as a physiologist is too well established throughout the whole medical world to admit of increase from any commendation of ours; but we should be doing injustice to our own feelings if we did not here express our admiration of his great intellectual powers, of his extensive learning, of the comprehensiveness of his views, of the quickness with which he seizes the important points and bearings of each subject, of the logical order in which he arranges his facts, and of the clearness and precision with which he explains and exposes his doctrines. Dr. Carpenter's various treatises are in fact models in their respective departments. It is their great and varied excellence which accounts for their unrivalled popularity. We can pay no higher compliment to the work before us than to say that it is equal in merit to the former productions of the author. This is equivalent to saying that it is, without question, the best manual or short treatise on physiology extant. Although designed for the student and framed expressly to meet his wants, it is a work, we will venture to say, that may be consulted with advantage by most physicians and surgeons, however learned.

### II. Mr. Fergusson's Surgery.

The first edition of this work has been so recently under our notice that we deem it unnecessary, at the present date, to make any lengthened addition to our former observations upon it. It is but justice, however, to state our belief that much time and labour must have been expended upon the present volume; and the author very modestly and ingenuously admits, in his preface, that "advantage has been taken of the suggestions of the reviewers to alter or amend whatever seemed faulty or defective." A close inspection shows that, besides the new matter, there has been a very careful revision of the original text throughout. To show that some of our own hints have not been thrown out in vain, we may refer to the author's notice of the entrance of air into the veins (p. 444) during operations. We thought that he had treated this dangerous event somewhat heedlessly, but he has now made amends; and although still adhering to his former opinions, he gives reasons for his peculiar views and quotes the authorities of Nysten, Velpeau, and Cormack, to prove that Bichat was wrong in his estimate of the quantity of air which was likely to induce fatal effects, much more being requisite than was imagined by that great authority.

The object of our author seems to be to confine himself as much as possible within the limits of what may be called *pure* surgery. There is certainly much pertaining to the ordinary practice of the surgeon which is not even alluded to in this work, and the author apologises for such deficiency by stating his opinion "that a 'Complete System of Surgery' should embrace all branches of professional knowledge, but that as each department has its separate authors and teachers, he has limited his work accordingly." Certainly, in a portable volume like that before us, it would have been impossible to have grasped all such topics comprehensively; and perhaps a tolerably fair criterion of the practical value of the particular selection adopted by the author, is the popularity which his work has so rapidly attained.

The following seem to be the principal novelties in the present edition:

The alterations in the first part of the volume, 'The Elements of Practical Surgery,' are more extensive than elsewhere. Some chapters of the former edition have been altered and transferred to other parts of the volume, and we observe the addition of a new one on the subjects of "boils, carbuncles, ulcers, scalds and burns, chilblains, and frostbite." We perceive, also, in this part of the work, that the author has not altogether neglected our former complaints as to the necessity of defining diseases as well as naming them; and although even yet we think him defective in this respect, we are sure that the additions which he has made will make his work more acceptable to all parties.

In the chapter on amputation (p. 149), our author gives reasons for entertaining a preference to "secondary" amputation rather than "primary" among civilians, and in this respect his doctrines are somewhat novel among systematic authors of the present day, the custom having so long prevailed of taking their data from military authorities. The views founded on the statistics of military practice have been somewhat modified, however, by Mr. Rutherford Alcock, and Sir George Ballingall; and the tables referred to by our author, as made up by Dr. Henry Reid from the books of the Royal Infirmary of Edinburgh, are exceedingly interesting, as showing the great success attending secondary operations of this description. The recent additions to operative surgery performed by Mr. Syme of Edinburgh, respecting amputations at the ankle and knee, are fully noticed by Mr. Fergusson; and we are pleased with the impartial manner in which he has considered these subjects. The bold operation of resection of the head and neck of the femur is given in detail; and the subject of staphyloraphy, for improvements in which the profession acknowledges itself indebted to our author, is here placed in a light at once novel and scientific. On the operation of harelip we perceive that Mr. Fergusson leans to the views expressed by Dubois, Malgaigne, Warren, and others, as to the advantages of early interference.

In conclusion, we will only add, that our former very favorable estimate of Mr. Fergusson's system is considerably enhanced by the examination of the present edition; and we can conscientiously recommend it to our readers as a work deserving a place in the library of every surgeon. To junior practitioners it may be said to be indispensable.

### III. MR. TAYLOR'S MEDICAL JURISPRUDENCE.

In our Journal for January, 1844, we noticed with high but well-deserved eulogy the first edition of this work. The rapidity with which a large impression has been disposed of is a satisfactory proof that the character we gave of it was just. As we expected, it has become truly *the manual* of both the medical and legal professions, and is regarded by all as the standard authority on its subject. The author, also, as we find from the public prints, is the person consulted, almost as a matter of course, in the more difficult medico-legal cases. In the present edition the work is greatly enlarged by the addition of new materials; and very important alterations and improvements are made in the former text. The following extract from the preface notices only a few of these changes:

"In the section on POISONING, I have thought it advisable to make a few alterations, in order that I might be enabled to introduce the principal discoveries made in Toxicology during the last two years. Among the changes may be enumerated,—the omission of some chemical processes for detecting poisons, and the substitution of others which appeared more simple,—a description of the methods employed for detecting poisons when absorbed into the tissues,—additions to the sections on arsenic, lead, copper and opium,—new facts connected with the smallest fatal doses of poisons, and the shortest periods for their operation,—cases illustrating the fatal effects which ensue from their external application;—and lastly, the strength of all medicinal preparations which contain poison. The chapter on prussic acid has been almost entirely rewritten; and in this edition will be found sections on poisoning by the Tartaric and Acetic acids, Lime, Phosphorus, Phosphoric acid, Chromate of Lead, Elder and Laburnum. . . . . .

"Numerous additions and alterations have been made in the chapters on Wounds and Infanticide. The sections on Rape, Pregnancy, Delivery and Abortion have also been enlarged; and some additions have been made to that of Insanity, including the changes respecting medical certificates, and the discharge of lunatics, under the new Lunacy Act."

To the various chapters on poisons, the author has appended a list of the medicinal preparations into the composition of which they enter, with the doses and the proportion of each poison in a given dose of the preparation. We perceive also that the rate at which each poison is retailed to the public, and the weights of some well-known popular measures, are mentioned, so that a medical practitioner may be able to give some information on these matters, when he is called upon to express an opinion regarding quantity or dose from the evidence of non-professional witnesses.

Among the additions to the section on infanticide, we may refer to the remarks made at p. 281, on the power of the lower class of females to exert themselves, and perform numerous acts requiring great strength and presence of mind, soon after delivery,—a question which frequently arises in cases of child murder.

In treating of *corpora lutea,* in the chapter on pregnancy, the author refers to the great changes in medical opinions, respecting the value of the evidence which the presence of these bodies is capable of furnishing. The views of Dr. Knox, Mr. Wharton Jones, and Professor Bischoff are briefly examined, and their bearing on practice pointed out.

In the chapter on Drowning some important remarks are made on the occurrence of accidental fractures of the vertebræ, part of which we here extract:

"*Fractures* are not often met with in the drowned as the result of accident during or after the act of submersion. Certain fractures likely to be followed by immediate death, may forbid the supposition of their having occurred after the act of submersion, and a careful examination of the body may show that they were not likely to have arisen from accident at or about the time of submersion. This point was raised in the case of *Reg.* v. *Kettleband,* (Nottingham Winter Ass. 1843,) where the prisoner was charged with the murder of his son, a boy aged ten years. The deceased was found dead in a pond soon after he had been seen healthy and well. An inquest was held, and, as usual, no inspection of the body was required by the coroner, and the jury were directed to return a verdict of 'found drowned.' An inspection was, however, subsequently made. The neck was observed to be very loose, and on further examination the processus dentatus was found to be separated from the atlas, and the ligaments were ruptured! The three medical witnesses who gave evidence at the trial, deposed that this displacement had caused death by compressing the spinal marrow,—that the injury had occurred during life,—that it was not likely to have been caused by accident from a fall into the water, as there was no mark of a bruise about the head, and the pond was proved to be small, with a soft muddy bottom. All agreed that such an injury was not likely to have arisen from a blow or a fall under any circumstances, because it required for its production, that the body should be fixed and the head forcibly rotated on the trunk. It was in itself sufficient to account for immediate death, and it could not occur by accident after death from any other cause. Hence it was inferred, 1, that death could not have been caused by drowning; 2, that it had resulted from the compression of the spinal marrow, by displacement of the second vertebra; and, 3dly, that this injury must have been intentionally produced by some person. Circumstances fixed the crime on the prisoner, and the jury returned a verdict of manslaughter; although the nature of the injury, admitting that it was not the result of accident, proved that the prisoner must have acted with a most cool and deliberate intention to destroy life! This case furnishes a serious commentary on the practice of certain coroners in denying the necessity for an inspection, and in directing what is called an open verdict of '*found drowned*,' where a body is taken out of water! It is an important medico-legal question, whether fractures of the *cervical vertebræ* can occur from accident alone, about the time of drowning. In the above case, the medical witnesses had probably good reasons for denying that the injury was accidental, although such an opinion cannot be always expressed merely from the absence of marks of violence on the head. Mr. South quotes the case of a man who threw himself into a river to bathe from a height of seven or eight feet, the water being only three feet deep. He rose to the surface, but fell back senseless. When he recovered his consciousness, the account he gave of the accident was, that he felt his hands touch the bottom of the river, but to save his head drew it violently back, upon which he lost all consciousness. He died in about ten hours, and on examination the back of the neck was much ecchymosed—the interspaces of the muscles were gorged with blood, and the vertebral canal filled with it. The body of the fifth cervical vertebra was broken across above the middle of its depth, and the two pieces were completely separated from the lateral parts. As there was no mark of contusion or dirt on the head, Reveillon, who reports the case, believes that the fracture arose from muscular action, and not from a blow received by striking the bottom; but this is doubtful. In another instance related by Mr. South, a sailor jumped headlong into the sea to bathe, a sail being spread three feet below the surface. He immediately became motionless, and died in forty-eight hours. The fourth and fifth cervical vertebræ were found extensively fractured, and the spinal marrow crushed and lacerated. Chelius's Surgery, Part vi., Fractures. In this case, the fracture must have resulted from contact with the water or the sail; but as the latter was freely floating, this would be a yielding medium, hence this serious injury may occur accidentally in cases in which we might not be prepared to look for it. (For an important medico-legal case, involving many questions connected with marks of violence on the drowned, see Ann. d'Hyg. 1839, ii. 195.)"

The other chapters on the various forms of Asphyxia have undergone considerable revision.

In the section on Insanity, which is much enlarged, the rules regarding certificates under the new act are fully stated for the guidance of practitioners, and a better arrangement has been adopted in the treatment of the subject of homicidal monomania. The causes, symptoms, and legal and medical tests are successively examined; then follow cases in illustration, including some of very recent occurrence. We here subjoin a portion of Mr. Taylor's general summary, and with it must conclude our very imperfect notice of this admirable treatise.

"A strange and unaccountable notion prevails in the public mind, that a homicidal lunatic is to be distinguished from a sane criminal by some certain and invariable symptoms or characters which it is the business of a medical witness to display, in evidence, and of a medico-legal writer to describe. But a perusal of the evidence given at a few trials will surely satisfy those who entertain this notion, that each case must stand by itself. It is easy to classify homicidal lunatics, and say that in one instance the murderous act was committed from a motive—i. e. revenge or jealousy ; in a second, from no motive but from irresistible impulse ; in a third, from illusion or a delusive motive—i. e. mental delusion ; in a fourth, from perverted moral feeling. This classification probably comprises all the varieties of homicidal insanity ; but it does not help us to ascertain in a doubtful case, whether the act was or was not committed under any of these psychological conditions. It will enable us to classify those who are acquitted on the ground of insanity, but it entirely fails in giving us the power to distinguish the sane from the insane criminal.

"According to M. Esquirol, whose views, more or less modified, are adopted by all other writers on the medical jurisprudence of insanity, the facts hitherto observed indicate *three degrees* of homicidal monomania.

"1. In the first, the propensity to kill is connected with absurd motives or actual delusion. The individual would be at once pronounced insane by everybody. Cases of this description are not uncommon, and they create no difficulty whatever. The accused are rarely allowed even to plead to the charge.

"2. In the second class, the desire to kill is connected with no known motive. It is difficult to suppose that the individual had any real or imaginary motive for the deed. He appears to be led on by a blind impulse which he resists and ultimately overcomes. (Case by Mr. Daniell, antè, p. 670.)

"3. In the third class, the impulse to kill is sudden, instantaneous, unreflecting, and uncontrollable (*plus forte que la volonté*). The act of homicide is perpetrated without interest, without motive, and often on individuals who are most fondly loved by the perpetrator. (Maladies Mentales, ii. 834.)

"These three forms differ from each other only in degree ;—the two first being strongly analogous to, but lighter modifications of the third. All the cases which came before M. Esquirol had three characters in common. An irritable constitution, great excitability, singularity, or eccentricity of character : and previously to the manifestation of the propensity, there was a gentle, kind, and affectionate disposition. As in other forms of insanity, there was some well-marked *change of character* in the mode of life. The period at which the disorder commenced and terminated could be easily defined, and the malady could be almost always referred to some moral or physical cause. In two cases it was traced to the result of puberty, and in four to the power of imitation. Attempts at suicide preceded or followed the attack : all wished to die, and some desired to be put to death like criminals. In none of the cases was there any motive for the act of homicide.

"M. Esquirol believes that there are well-marked distinctions between this state and that of the sane criminal. Among these he enumerates, 1st, the want of accomplices in homicidal monomania. 2d. The criminal has *always* a motive—the act of murder is only a means for gratifying some other more or less criminal passion ; and it is almost always accompanied by some other wrongful act. The contrary exists in homicidal monomania. 3d. The victims of the criminal are those who oppose his desires or his wishes :—the victims of the monomaniac are among those who are either indifferent, or are the most dear to him. 4th. The criminal endeavours to conceal, and if taken, denies, the crime ; if he confesses it, it is only with some reservation and when circumstances are too strong against him ; but he commonly denies it to the last moment. It is the reverse with the monomaniac." (pp. 673-4.)

This section has also, we observe, an addition to it "on the Civil and Criminal Responsibility of the Deaf and Dumb."

